# The effects of long-term endurance training on the immune and endocrine systems of elderly men: the role of cytokines and anabolic hormones

**DOI:** 10.1186/1742-4933-3-9

**Published:** 2006-08-25

**Authors:** Milton Hideaki Arai, Alberto JS Duarte, Valéria Maria Natale

**Affiliations:** 1 Disciplina de Clínica Geral do Hospital das Clínicas da Faculdade de Medicina da Universidade de São Paulo, Brazil; 2 Laboratório de Investigação Médica (LIM-56) da Faculdade de Medicina da Universidade de São Paulo, Brazil

## Abstract

**Background:**

a decline in immune and endocrine function occurs with aging. The main purpose of this study was to investigate the impact of long-term endurance training on the immune and endocrine system of elderly men. The possible interaction between these systems was also analysed.

**Results:**

elderly runners showed a significantly higher T cell proliferative response and IL-2 production than sedentary elderly controls. IL-2 production was similar to that in young adults. Their serum IL-6 levels were significantly lower than their sedentary peers. They also showed significantly lower IL-3 production in comparison to sedentary elderly subjects but similar to the youngs. Anabolic hormone levels did not differ between elderly groups and no clear correlation was found between hormones and cytokine levels.

**Conclusion:**

highly conditioned elderly men seem to have relatively better preserved immune system than the sedentary elderly men. Long-term endurance training has the potential to decelerate the age-related decline in immune function but not the deterioration in endocrine function.

## Background

Human immune function undergoes adverse changes with aging, potentially leading to an increased risk of infections, a greater occurrence of autoantibodies and lymphoproliferative disorders, and a greater morbidity and mortality in the elderly [1]. Of the various components of the immune system, T cells are the most sensitive to the effects of aging [2]. Mitogen-induced T cell proliferation is usually reduced, and this may be the result from disruption of the well-balanced network of regulatory cytokines. The interleukin-2 (IL-2) production tends to diminish with age [3].

The endocrine system also suffers from senescence [4]. Substantial decline occurs in the hormone levels of at least three endocrine axes: hypothalamic-pituitary-gonadal, hypothalamic-pituitary-adrenal (HPA) and growth hormone-insulin-like growth factor I. Among them, the HPA axis is the one that best integrates the neuroendocrine and immune systems. The relationship between dehydroepiandrosterone sulphate (DHEAS) and interleukin-6 is described. There have been few reports concerning this field in the elderly [5]

The question arises whether regular physical activity can correct the deleterious effects of aging on the human immune and endocrine systems. There is growing evidence that long-term conditioning may be associated with improved immune functioning in the elderly [6], and physical activity has been reported to affect the endocrine profile in aged men [7,8]. No previous studies have examined the impact of long-term training on the immune and endocrine system concomitantly.

The purpose of the present investigation was to examine the effects of long-term endurance training on the T cell proliferative response, broad cytokine profile and serum hormone levels in elderly men and to correlate the anabolic hormones with cytokines.

## Results and discussion

Preservation of immunological functions is keenly needed to avoid disease and consequently for the improvement of quality of life in the elderly. Several methods to improve the immune system have been tested but their effectiveness is generally controversial [9]. Among them, regular physical exercise has been tested in limited studies and has been proposed as an effective intervention in the aged [6].

**Figure 1:**
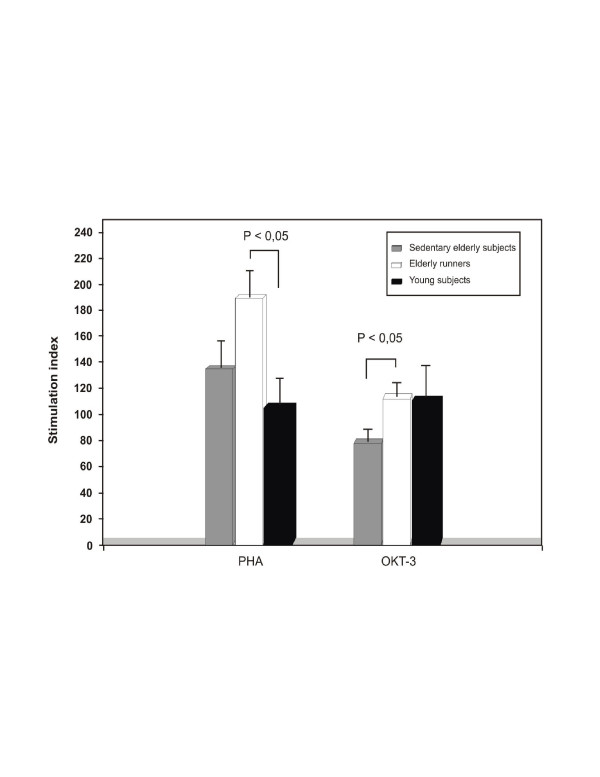
PBMC proliferative response to mitogens (PHA and OKT-3). Elderly runners showed a trend to a higher response to PHA and a significantly higher response to OKT-3 than sedentary elderly subjects. They also showed a higher response to PHA than young subjects but not to OKT-3. Vertical bars indicate ±S.E.M. from mean value.

In general, cross-sectional studies examining highly active elderly subjects have demonstrated higher *in vitro *T cell responses to polyclonal stimulation when compared to sedentary elderly subjects [10,11]. This was corroborated by our study that showed a higher mitogen-stimulated peripheral blood mononuclear cells (PBMC) proliferation in elderly runners in comparison to their sedentary peers (Figure 1). However, prospective human studies using short training programs (≤24 weeks) have failed to demonstrate consistent improvement of proliferative responses in older adults [10,12,13]. Thus regular physical activity for many years may help to counter the age-related decline in the potential of T cells to proliferate.

Very little is known about the effect of exercise training on the cytokine profile of elderly humans. Shinkai et al. [11] showed greater production of IL-2, interferon (IFN)-γ and IL-4 in elderly runners when compared to sedentary peers. Nevertheless, twelve week of resistance strength training did not induce changes in IL-1β, tumor necrosis factor (TNF)-α, IL-2 or IL-6 production in elderly subjects [12]. Jankord and Jemiolo showed lower levels of serum IL-6 and higher levels of serum IL-10 in the very active group compared with the less active group [14].

**Figure 2:**
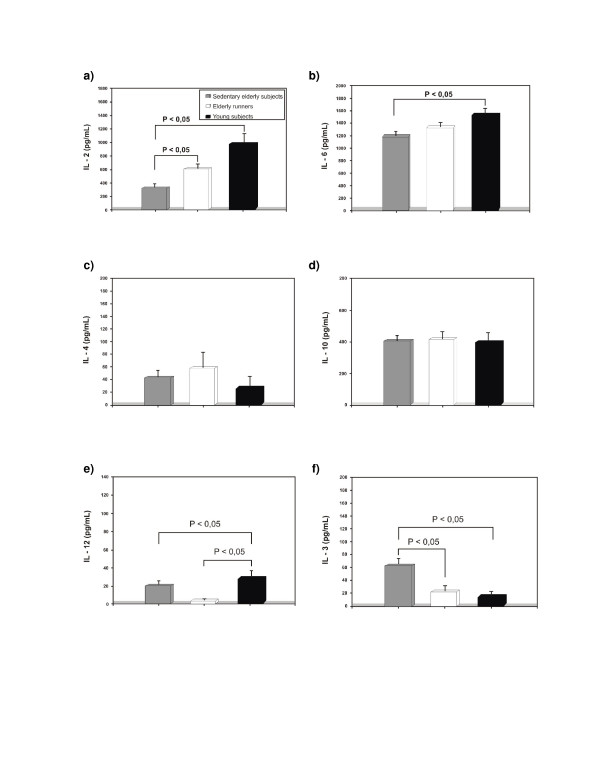
Production of cytokines by PHA-stimulated PBMC. Results represent the mean ± S.E.M.

Decreased IL-2 production of PBMC probably reflects a major mechanism by which immune responses are decreased with increasing aging. IL-2 is a key component for the generation of any immune response due to the major role of T cells in regulating both T and B cell responses. Our elderly runners showed higher IL-2 production than their sedentary peers and similar production to the young subjects (group effect: p =< 0.001) (Figure 2a). This finding may be the most important signal showing the immunological benefits of chronic exercise. It could be associated with delay in immunosenescence. Higher IL-2 production may be associated with higher T cell proliferative response.

In contrast to IL-6 production (group effect: p = 0.046) (Figure 2b), serum IL-6 levels were significantly lower in elderly runners than in sedentary elderly subjects (Table 1). Recent study with fewer number of very healthy older males has also shown association of higher volume of regular physical activity with decreased IL-6 levels [14]. It has been proposed that serum IL-6 levels may be a good overall biomarker of health in aging because levels are correlated with functional status and prospectively with morbidity and mortality [15-17]. It is possible that regular exercise training may protect against future disability and mortality by attenuating low grade inflammatory process that occur during aging [18].

**Table 1:** Levels of cytokines (pg/ml) in serum of elderly subjects (mean ± S.E.M.)

Cytokines	Elderly runners	Sedentary controls
IL-2	90.1 ± 3.4	101.9 ± 5.7
IL-3	3.8 ± 1.9	15.2 ± 4.7*
IL-4	3.4 ± 3.4	Not detectable
IL-6	0.6 ± 0.3	3.7 ± 0.9**
IL-10	0.06 ± 0.04	1.73 ± 0.69
IL-12	11.5 ± 1.5	20.5 ± 2.5**

*p < 0.05

**p < 0.01

In our study, IL-4 and IL-10 (group effect: p = 0.149 and p = 0.934 respectively) were not altered in elderly runners compared to the sedentary group (Figure 2c,d). It is possible that long-term aerobic training does not affect the Th2-like cytokine response in aged men.

In spite of a higher proliferative response and IL-2 production in relation to their sedentary counterparts, the elderly runners did not show any difference in IL-12 production (group effect: p =< 0.001) (Figure 2e). Chronic exercise seem to change T cells function but its effects on monocytes, macrophages and dendritic cells that secrete IL-12 are little known [19]. As a non-specific mitogen phytohemagglutinin-A (PHA) was used to stimulate these cells, future comparison of IL-12 productions induced with specific antigen (bacterial lipopolysaccharide) and of kinetics of release of this cytokine with both stimuli could be done. Since IL-12 is an inducer of Th1 cell generation and upregulates IFN-γ production [20], the measurement of this cytokine could be also helpful to elucidate this finding.

The effects of exercise training on IL-3 production in elderly people have not been previously investigated. This interleukin is preferentially produced by T cells and it functions as a link between the immune and hemopoietic systems, stimulating the generation and the function of blood cells, specially the pluripotential hemopoietic stem cell and its derivatives [21]. Our active elderly subjects showed lower production and serum levels of IL-3 than their sedentary peers and similar production in relation to young subjects (group effect: p =< 0.001) (Figure 2f and Table 1). This suggests that long-term training may counteract the effect of aging on synthesis of this interleukin and maybe facilitates the homeostasis of hemopoietic system.

Insulin-like growth factor-I (IGF-I), growth hormone (GH), testosterone and dehydroepiandrosterone-sulfate have been reported to have anabolic effects on muscle and bone mass and to be associated with a globally increased physical and psychological well-being in elderly people [8]. Circulating levels of these hormones usually decrease with male aging [4] but it is unclear whether this decrease is an unavoidable effect of aging itself or reflects the influences of modifiable external factors such as lifestyle. It is still uncertain whether habitual moderate physical activity may counteract the age-associated reduction in blood levels of endogenous anabolic hormones.

Few studies have focused on the relationship between these hormones and regular physical activity in older subjects. A recent study showed association of regular moderate physical activity with higher levels of DHEAS and IGF-I in aging men [8]. In another study significant positive correlations between DHEAS and energy expenditure in both light physical activities and moderate intensity sports were found for elderly women but not for men [7]. In that study, the DHEAS levels of elderly women were also correlated with maximal oxygen comsumption (VO_2 _max). On the other hand, Abbasi et al. [22] reported that the DHEAS values of older men (but not of older women) were associated with VO_2 _max but not with energy expenditure in physical activities. Others demonstrated a small increase in fasting level of IGF-I in men [23] and women [7]. However, some studies have found no relation between regular physical activity and serum/plasma levels of IGF-I or GH [24-26]. Studies on older subjects have not shown significant chronic effects of high-intensity exercise on blood testosterone levels [25,26].

In the present study, the levels of endogenous anabolic hormones of elderly runners were similar to those of their sedentary counterparts (Table 2). We also did not find a correlation between DHEAS concentrations and VO_2 _max. Apart from physical activity and aging, hormone concentrations can also be affected by a wide variety of factors including smoking, dietary habits, health status (including immunological status) and body composition. For this reason, when we selected the volunteers, we adopted very strict exclusion criteria, like the SENIEUR protocol [27] and some nutritional parameters (data not shown), in order to avoid the possibility that hormone concentrations might reflect concurrent illnesses or lifestyle variables other than physical activity. Our results indicate that high levels of regular endurance exercise do not seem to prevent even partially the somato-, gonado- and adreno-senescence.

**Table 2:** Regular physical activity and serum hormone concentrations in elderly men (mean ± S.E.M.)

Hormones	Elderly runners	Sedentary controls
DHEA-S (μg/dl)	79 ± 6	92 ± 14
Free testosterone (pmol/l)	258.4 ± 15.5	271.6 ± 19.1
Total testosterone (ng/dl)	529 ± 41	456 ± 36
GH (μg/l)	0.44 ± 0.12	0.68 ± 0.32
Cortisol (μg/dl)	13.7 ± 0.6	13.2 ± 0.8
Cortisol/DHEA-S	0.19 ± 0.01	0.19 ± 0.02

No statistical differences were observed between groups

Possible links between endocrinosenescence and immunosenescence have been studied [5,28-30]. The HPA axis has gained more attention because DHEA(S) have shown some immunomodulatory effects, specially on cytokines [5]. Studies showed that DHEA inhibited the stimulated production of IL-6 by human PBMC [28] and spontaneous production of this interleukin by human splenocytes [29]. They also noted an inverse relationship between DHEA(S) and serum IL-6 during aging in humans. However divergent results have also been obtained [30], indicating that the relationship between IL-6 and DHEA(S) in man is more complex than perhaps hitherto appreciated.

In the present study we did not find any correlation between DHEAS and serum IL-6 levels in elderly subjects. Poor correlation was observed between DHEAS and other cytokines measured. To our knowledge, this is the first study that tested the correlation between DHEAS and Th1 and Th2 type cytokines concomitantly in elderly men. No correlation was found between other hormones and cytokines. The contribution of hormones to immunosenescence does not seem to be so clear at least between 60 and 80 years old.

We conclude that highly conditioned elderly men seem to have relatively better preserved immune system than the sedentary elderly men. Regular endurance exercise can correct some detrimental immune changes of aging but does not seem to prevent endocrinosenescence.

## Methods

### Study subjects

All subjects gave written consent prior to their inclusion in the study.

The three test groups comprised 20 older recreational runners (age range: 61–80 years), 20 age-matched sedentary controls (age range: 60–75 years), and 10 young sedentary controls (age range: 23–34 years). The mean ages (±SEM) for the three groups were: 66.7 ± 1.0, 65.8 ± 0.9, and 26 ± 1.8 years, respectively. Only men were studied.

The active elderly subjects reported running an average of 54 ± 2 min.d^-1^, 4.3 ± 0.2 d.wk^-1^, covering a weekly distance of 38.7 ± 2.6 km (range: 25–60 km). Subjects had maintained this level of exercise for 23 ± 2 years.

The sedentary subjects were not engaged in any kind of physical activity of ≥15 minutes duration more than 3 times per week for the previous 2 years.

The maximal oxygen consumption (VO_2 _max) of these volunteers was measured. VO_2 _max was assessed by progressive and continuous testing on a treadmill until exhaustion, according to the Bruce protocol [31]. All subjects reached their age-predicted maximal heart rate and maximal respiratory quotient (RQ ≥ 1.10). Oxygen uptake and ventilation were measured using a Vmax Series 229 metabolic cart (SensorMedics, Yorba Linda, CA-USA). The elderly runners presented a 52% higher VO_2 _max (38.5 ± 1.1 ml.kg^-1^.min^-1^) than the sedentary elderly subjects (25.5 ± 0.8 ml.kg^-1^.min^-1^), matching the level seen in sedentary young subjects (36.6 ± 2.1 ml.kg^-1^.min^-1^).

Subject selection was performed according to the SENIEUR protocol [26]. Exclusion criteria included: systemic diseases, such as cardiac, liver, kidney and bone marrow disorders, diabetes, acute and chronic inflammatory conditions, clinical depression, neurodegenerative disease, anemia, leucopenia, alcoholism, and undernutrition. Subjects were excluded if they smoked and if they were taking any medications, vitamins or food supplements known to affect immune function. Recent (<3 months) surgery, infection, or vaccination and previous history of cancer or immune disorders were also considered exclusion criteria.

### Laboratory methods

Blood specimens were collected from all subjects in the seated position between 7:00 and 8:00 h after resting for a minimum of 30 minutes and abstaining from all food, beverages (except water) for at least 8 h, and vigorous physical activity for at least 48 h.

#### Preparation of peripheral blood mononuclear cells (PBMC) and proliferative response

PBMC suspensions were prepared from heparinized venous blood using a Ficoll-Hypaque gradient, washed with RPMI 1640 medium, and resuspended in RPMI supplemented with 10% type AB human serum (Sigma, St Louis, MO-USA). Two × 10^6^/ml cells per well were cultivated in triplicate in flat-bottomed 96-well plates for 3 days, at 37°C and 5% CO_2_, in the presence of 2.5 μg/ml phytohemagglutinin-A (PHA) (Difco Laboratories, Detroit, MI-USA) and 5 μg/ml anti-CD3 monoclonal antibodies (OKT3). Cultures containing no mitogens were used as controls. The cultures were pulsed with 0.5 μCi per well of [^3^H] thymidine (Amersham Pharmacia Biotech, Buckinghamshire, England) 18 hours prior to harvesting, and the amount of radioactivity incorporated was determined with a scintillation counter (1205 Betaplate, Wallac Oy, Turku, Finland). Proliferation values are presented in the form of stimulation index, calculated from the ratio radioactivity in stimulated culture to radioactivity in non-stimulated culture.

#### Cytokine production and serum cytokines

Culture supernatants were harvested by centrifugation after stimulation with PHA for 24 h, as described above. Supernatants were stored at -70°C until analysis in duplicates using a solid-phase sandwich ELISA kit (Quantikine; R & D Systems, Minneapolis, MN-USA).

Levels of IL-2, IL-3, IL-4, IL-6, IL-10 and IL-12 were measured. Results were expressed as pg/ml. The detection limits were: <0.7 for IL-2, <7.4 for IL-3, <10 for IL-4, <0.7 for IL-6, <3.9 for IL-10 and <5.0 for IL-12.

Serum cytokines levels were also determined in elderly subjects.

#### Measurement of serum hormone levels

Dehydroepiandrosterone sulfate was assayed by radioimmunoassay and growth hormone was assayed by immunofluorometric assay. Testosterone was assayed using an electrochemiluminescence immunoassay and free testosterone value was calculated from total testosterone and immunoassayed SHBG concentrations (SHBG, sex hormone-binding globulin). Cortisol was assayed using a fluoroimmunoassay. The intraassay and interassay coefficients of variation were below 7.5% and 8.0% in each test, respectively.

Serum hormone levels were measured only in elderly subjects.

### Statistics

Statistical analysis and power calculations were performed using the SigmaStat software (Jandel Scientific, San Rafael, CA-USA). Results are expressed as mean ± SEM. Group comparisons were made using a one-way ANOVA or Kruskal-Wallis test. For *post-hoc *multiple comparisons, a Tukey test or a Dunn procedure were made. Comparisons of the values between two groups were performed by Student t-test or Mann-Whitney rank sum test depending on the normality of the distribution curves. The correlation between two variables was analyzed by the Pearson correlation coefficient. The level of significance was set at p < 0.05.
